# The Microbiota Profile Analysis of Combined Periodontal-Endodontic Lesions Using 16S rRNA Next-Generation Sequencing

**DOI:** 10.1155/2021/2490064

**Published:** 2021-11-16

**Authors:** Ping Sun, Zhiyong Guo, Daiping Guo, Jian Wang, Tingting Wu, Tingjun Li, Jiannan Liu, Xinhua Liu

**Affiliations:** ^1^The First People's Hospital of Jinzhong, Jinzhong City, 030600 Shanxi Province, China; ^2^Department of Oromaxillofacial-Head & Neck Oncology, Shanghai Ninth People's Hospital, College of Stomatology, Shanghai Jiao Tong University School of Medicine, National Clinical Research Center for Oral Diseases, Shanghai Key Laboratory of Stomatology & Shanghai Research Institute of Stomatology, Shanghai 200011, China

## Abstract

**Objective:**

The primary aim of this investigation was to analyze the microbiome in patients with combined periodontal-endodontic lesions.

**Method:**

Patients with loose and/or painful teeth referred for treatment from March 2020 to December 2020 in the First People's Hospital of Jinzhong were recruited. Samples were collected from teeth diagnosed as chronic periodontics (PE), ulcerative pulpitis (PU), and retrograde pulpitis (RE). Genomic DNA was extracted. The quantitative polymerase chain reaction, targeting the 16S ribosomal RNA (rRNA), was adopted for the quantification of bacteria. Then, the V3-V4 hypervariable regions of the 16S rRNA gene were amplified and subjected to next-generation sequencing. The statistical analysis was performed by R software (V3.5.1).

**Results:**

A total of 57 qualified samples were collected from 48 patients and analyzed (7 PE, 21 PU, and 19 RE). By linear discriminant analysis effect size, *Kingella* and *Barnesiella* were significantly increased in the periodontal pocket of retrograde pulpitis (RE-PE), compared with PE. The relative abundance of *Clostridiales Incertae Sedis XI*, *Fusobacteriaceae*, *Fusobacterium*, *Parvimonas*, *Micrococcaceae*, and *Rothia* was significantly increased in the pulp of retrograde pulpitis (RE-PU) than PU and RE-PE. *Prevotella*, *Leptotrichia*, *Porphyromonas*, *Streptococcus*, and *Fusobacterium* are consistently at a high abundance, across PU, RE-PE, and RE-PU.

**Conclusion:**

The current study highlighted the evidence that a specific microbial community is associated with the occurrence of retrograde pulpitis. The microenvironment of the root canal and pulp chamber will select microbiota. This study offered insights into the pathogenesis of retrograde pulpitis.

## 1. Introduction

Firstly described by Simring and Goldberg in 1964 [1], combined periodontal-endodontic lesions have been classified into three categories based on the primary site [2]: (a) endodontic lesions with secondary periodontic involvement (retrograde pulpitis), (b) periodontic lesions with secondary endodontic involvement (retrograde periodontics), and (c) “TRUE” combined lesions. The periodontium and endodontium are tightly bonded embryonically, anatomically, and functionally, making combined periodontal-endodontic lesions a dilemma to diagnose and treat thoroughly [3, 4]. It has been widely accepted that the anatomical interconnection of periodontium and endodontium with pathogenic microorganism transmission is accused of inducing the former two types of diseases as above [5]. Several researchers have found the common microbial composition between infected root canals and advanced periodontitis through the traditional bacterial culture method [6–9]. However, restricted to the conventional research technique, only cultivable microbes could be detected, and the panoramic difference between infected root and derived periodontitis is still not known.

Being different from caries-caused pulpitis [10, 11], the infections of retrograde pulpitis are thought to be derived from those microorganisms that exist deep in the periodontal pocket [4, 12]. However, not all teeth with chronic periodontitis will progress into retrograde pulpitis eventually, inspiring us that apart from anatomical factors of root canals, discrepant microbiome profiles of periodontal pockets may also lead to the onset of pulpitis or not.

Several approaches have been applied in identifying pathogens. Culture and biochemical testing, with the advantage of low cost, is considered the golden standard of pathogen identification [13]. The main limitation of the culture and biochemical testing is that not all pathogen is cultivable. Next-generation sequencing (NGS), developed in 2005, can sequence billions of DNA fragments independently and simultaneously [14]. The characteristic of NGS is the ability to identify unculturable bacteria, shorter turnaround time, and more accurate results [15]. Thus, NGS has been widely adopted in the investigation of the association between microorganisms and oral diseases [16–19].

The principal aim of the present study is to compare the microbiome composition of chronic periodontitis (PE), ulcerative pulpitis (PU), and retrograde pulpitis (RE). The 16S rRNA gene sequencing technique was adopted to analyze the composition of the microbial community, aiming to elucidate the possible driving force for the development of periodontitis into retrograde pulpitis and to provide a theoretical basis for the early intervention of the disease.

## 2. Materials and Methods

### 2.1. Collection of Clinical Samples and Sampling Procedures

Patients with loose and/or painful teeth referred for treatment from March 2020 to December 2020 in the First People's Hospital of Jinzhong were screened and eligible patients were included. Teeth with probing depth (PD) ≥ 6 mm, attachment level (AL) ≥ 5 mm, looseness ≥ II°, and obvious alveolar bone resorption were diagnosed as PE. A tooth that had extensive caries lesions that led to pulp exposure without root canal treatment history and sensitivity to cold and or heat tests was diagnosed as PU. The diagnosis of RE included classical periodontics symptoms and the following criteria: (1) caries-free and intact crown; (2) spontaneous, cold, and/or heat-evoked localized or diffused pain; (3) no history of trauma; and (4) lack of periapical lesion radiographically with no sinus tracts. The summary of grouping abbreviations is presented in Table 1.

Exclusion criteria include (1) long-term medication history or antibiotics are taken within the past 3 months; (2) women during pregnancy, lactation, or menstruation; (3) have periodontal and endodontic treatment during the past 3 months; (4) history of orthodontic treatment; and (5) smoking history.

The study was approved by the Ethics Committee of the First People's Hospital of Jinzhong (2020-006-01). Written informed consent was obtained from each patient.

### 2.2. Microbial DNA Extraction and Sequencing

Genomic DNA was extracted from samples by using the DNeasy Blood & Tissue Kit (Qiagen, Germany) according to the manufacturer's instructions. DNA quality was evaluated by absorbance ratios of A260 to A280 using spectrophotometry (NanoDrop8000, Thermo Scientific). Only DNA samples with a ratio of A260 to A280 higher than 1.8 were recognized as qualified samples and used for subsequent analysis. The hypervariable V3-V4 region of the bacteria 16S ribosomal RNA genes was amplified using the primer pair 5′-CCTACGGGRSGCAGCAG-3′ (forward primer) and 5′-GGACTACVVGGGTATCTAATC-3′ (reverse primer), with the following PCR conditions: 95°C for 3 min, followed by 30 cycles at 98°C for 20 s, 58°C for 15 s, and 72°C for 20s and a final extension at 72°C for 5 min. PCR reactions were performed in 30 *μ*L mixture containing 15 *μ*L of 2× KAPA Library Amplification ReadyMix, 1 *μ*L of each primer (10 *μ*M), 50 ng of template DNA, and ddH_2_O.

Sequencing was performed according to a previously described protocol [20, 21]. In brief, amplicons were extracted from 2% agarose gels and purified using the AxyPrep DNA Gel Extraction Kit (Axygen Biosciences, Union City, CA, USA) according to the manufacturer's protocols and quantified using Qubit®2.0 (Invitrogen, USA). All quantified amplicons were pooled to equalize sequencing concentrations using Illumina MiSeq/PE250 (Illumina, Inc., CA, USA). The paired-end reads of 425 bp were overlapped on their 3′ ends for concatenation into original longer tags by using PANDAseq (https://github.com/neufeld/pandaseq, V2.9). DNA extraction, library construction, and sequencing were performed at Realbio Genomics Institute (Shanghai, China). Operational taxonomic units (OTUs) were clustered with 97% similarity by using USEARCH (V7.0.1090). Each representative tag was assigned to taxa by RDP Classifier (http://rdp.cme.msu.edu/). QIIME (V1.9.1) and R (V3.5.1) were also used to analyze and profile differences of results.

### 2.3. Statistical Analysis

The statistical analysis of alpha diversity, beta diversity, and statistically significant differences analysis was performed by R (V3.5.1) and QIIME (V1.9.1). Only average relative abundance > 0.1% would be counted in the statistical analysis. Values of *P* < 0.05 were considered to be statistically significant.

## 3. Results

### 3.1. Subject Characteristics

A total of 48 patients fulfilling the criteria were recruited. Among them, 23 (47.9%) were males and 25 (52.1%) were females. The average age of included patients was 45.2 years old (range 12-76 years old). Most of the teeth were molar (71.7%). The characteristics of recruited patients are shown in Table 2.

### 3.2. 16S rRNA Gene Sequencing

We sequenced 16S rRNA gene amplicons from 57 qualified samples (PE 17, PU 21, and RE 19). For RE, 10 samples were collected from the periodontal pocket (RE-PE) and 9 samples were collected from the pulp (RE-PU). All samples passed quality control. The number of total valid reads from 16S rRNA was 2.04 × 10^6^, ranging from 29,733-38,987 reads (mean 35,763 reads). Using a 97% similarity level, a total of 1163 OTUs were detected. The detailed distribution of OTUs is shown in Figure S1.

### 3.3. The Differences of Microbial Community Structure among the PE, RE-PE, and RE-PU Groups

**Figure 1:**
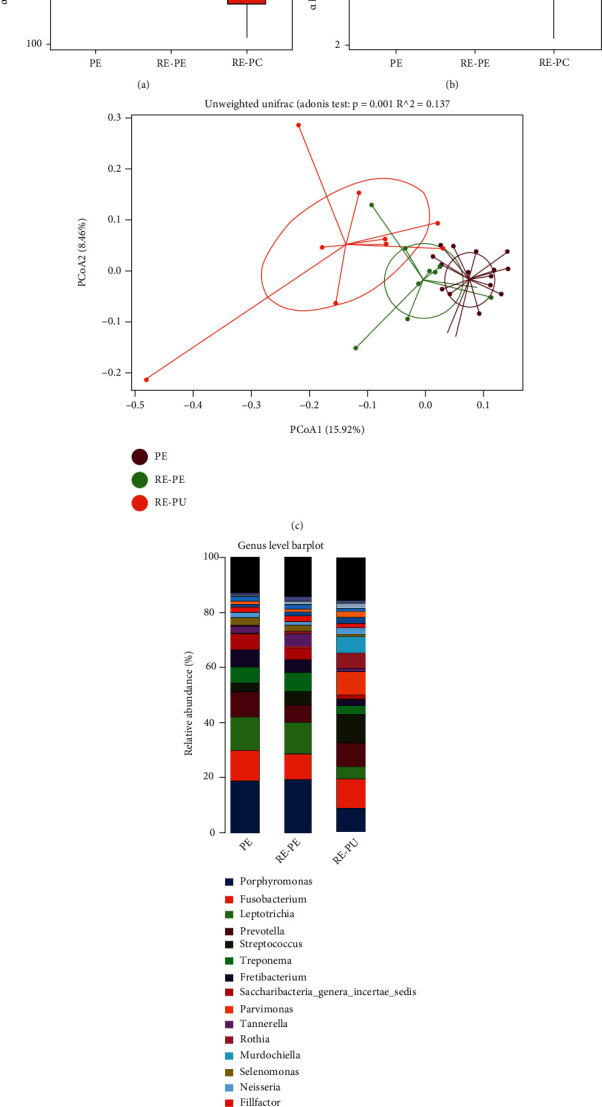
The analysis of microbial community structure of PE, RE-PE, and RE-PU groups. (a, b) The boxplot diagrams show alpha diversity index among PE, RE-PE, and RE-PU. (c) The plot of principal coordinates analysis (PCoA) shows intergroup distances by 2 principal coordinates. (d) The relative abundance of bacterial taxa at the genus level of PE, RE-PE, and RE-PU.

**Figure 2:**
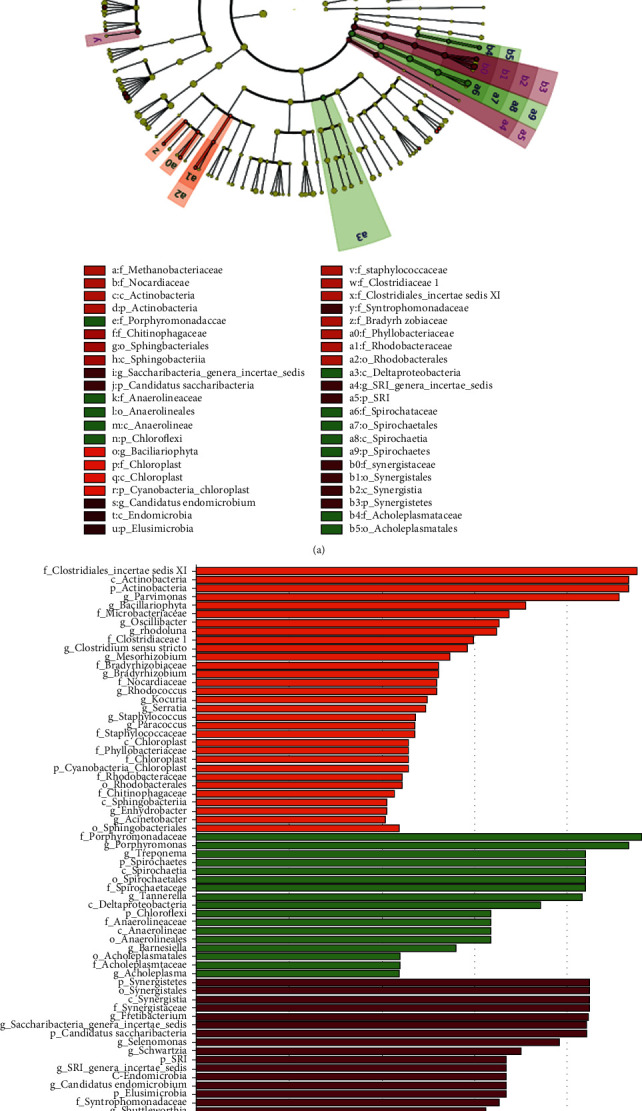
The linear discriminant analysis (LDA) effect size (LEfSe) profiles of PE, RE-PE, and RE-PU. (a) Cladograms indicating the phylogenetic distribution of bacterial lineages among 3 groups. The phylum, class, order, family, and genus levels are listed in order from inside to outside of the cladogram, and the labels for levels of order, family, and genus are abbreviated by a single letter. (b) LDA along with effect size measurements was applied to present the enriched bacterial genera of each group.

The alpha diversity analysis included community richness (Chao1 diversity index) and community evenness (Shannon diversity index). In general, the RE-PU yielded the lowest community richness, followed by RE-PE and PE (*P* < 0.05) (Figure 1(a)). For the community evenness, RE-PU was significantly lower than PE (Figure 1(b)). The beta-diversity was analyzed to depict divergence among PE, RE-PE, and RE-PU groups. The principal coordinates analysis (PCoA) diagram showed that three groups were relatively independent indicating significantly different microbial communities (Adonis analysis, *P* = 0.001) (Figure 1(c)). The distribution of the top 20 genera is depicted in Figure 1(d). Compared with RE-PE and PE, *Porphyromonas*, *Leptotrichia*, *Saccharibacteria*, and *Selenomonas* were significantly reduced in RE-PU, while *Streptococcus*, *Parvimonas*, *Rothia*, and *Murdochiella* were significantly rich in RE-PU. The linear discriminant analysis (LDA) effect size (LEfSe) analysis was adopted to find the species with an abundance that is significantly different among multiple groups. As shown in Figure 2, a total of 32 genera (represented by *Clostridiales Incertae Sedis XI*, *Actinobacteria*, and *Parvimonas*) were significantly abundant in RE-PU, 17 genera (represented by *Porphyromonadaceae*, *Porphyromonas*, *Treponema*, and *Spirochaetes*) were abundant in RE-PE, and 20 genera (represented by *Synergistetes*, *Synergistales*, *Synergistia*, and *Synergistaceae*) were abundant in PE.

### 3.4. The Differences in Microbial Community Structure among the PU, RE-PU, and RE-PE Groups

**Figure 3:**
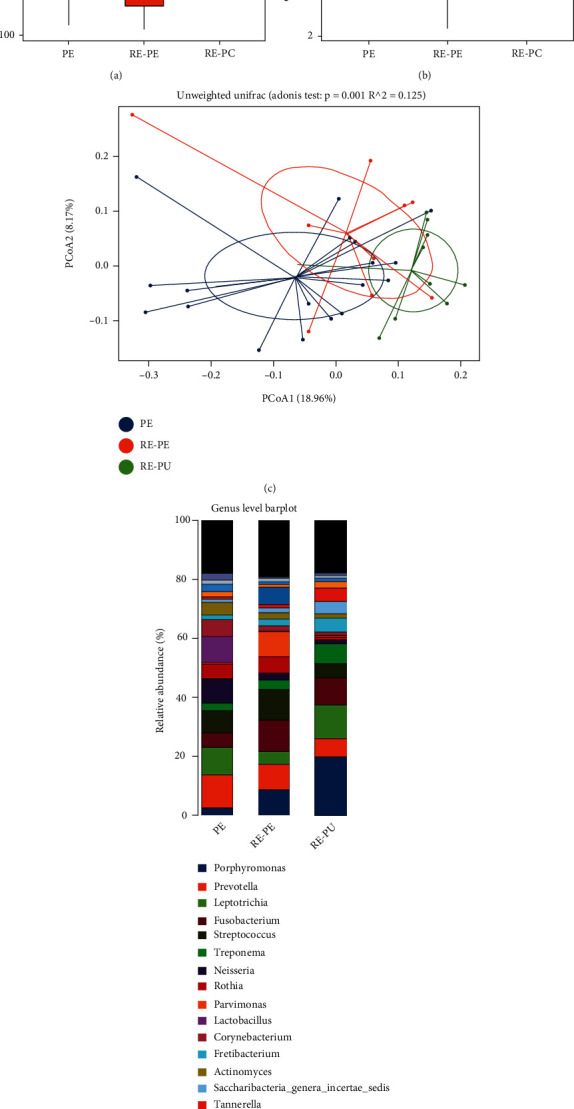
The analysis of microbial community structure of PU, RE-PU, and RE-PE groups. (a, b) The boxplot diagrams showed alpha diversity index among PU, RE-PU, and RE-PE. (c) The plot of principal coordinates analysis (PCoA) showed intergroup distances by 2 principal coordinates. (d) The relative abundance of top 20 bacterial taxa at the genus level for PU, RE-PE, and RE-PU.

**Figure 4:**
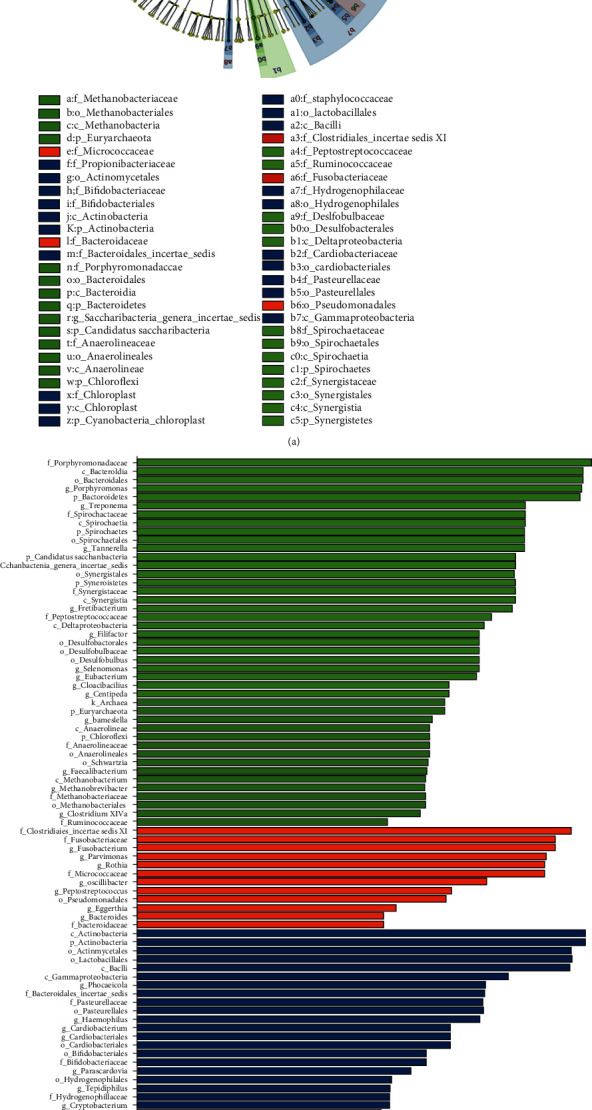
The linear discriminant analysis (LDA) effect size (LEfSe) profiles of PU, RE-PU, and RE-PE. (a) Cladograms indicating the phylogenetic distribution of bacterial lineages among 3 groups. The phylum, class, order, family, and genus levels are listed in order from inside to outside of the cladogram, and the labels for levels of order, family, and genus are abbreviated by a single letter. (b) LDA along with effect size measurements was applied to present the enriched bacterial genera of each group.

Alpha diversity showed higher richness and evenness in RE-PE, while there are no statistically significant differences between PU and RE-PU (Figures 3(a) and 3(b)). PCoA diagram indicated three relatively independent groups (Figure 3(c)), testified by Adonis (*P* = 0.001), Anosim (*P* = 0.003), and MRPP (*P* = 0.002) analyses. Genera of the top 20 relative abundances were marked in Figure 3(d). In detail, the abundance of *Leptotrichia*, *Selenomonas*, and *Capnocytophaga* were significantly lower in RE-PU than PU and RE-PE. Meanwhile, the abundance of *Streptococcus*, *Parvimonas*, and *Murdochiella* was significantly higher in RE-PU, compared with PU and RE-PE. LEfSe analysis filtered genera with a significant difference in relative abundance are shown in Figure 4. In specific, RE-PE was rich in *Porphyromonadaceae*, *Bacteroidia*, *Bacteroidales*, *Porphyromonas*, and *Bacteroidetes*. For RE-PU, the relative abundance of *Clostridiales Incertae Sedis XI*, *Fusobacteriaceae*, *Fusobacterium*, *Parvimonas*, *Micrococcaceae*, and *Rothia* was significantly increased, while the most selectively enriched abundant genera for PU were *Actinobacteria*, *Actinomycetales*, *Lactobacillales*, and *Bacilli*.

### 3.5. The Function Prediction of Microbial Community

**Figure 5:**
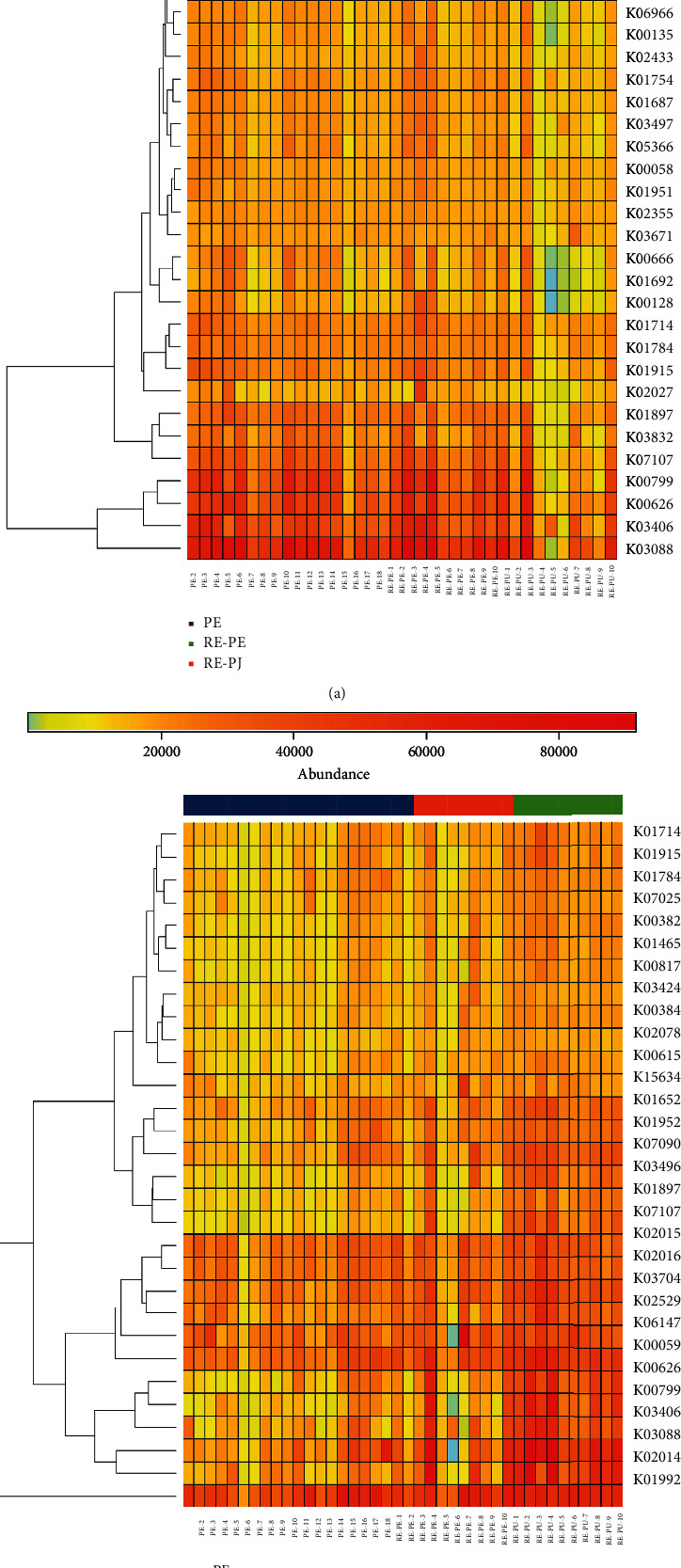
The heat map of Kyoto Encyclopedia of Genes and Genomes (KEGG) pathway categories based on differently detected genera of (a) PE, RE-PE, and RE-PU and (b) PU, RE-PE, and RE-PU.

The function of a differently detected microbial community was predicted using KEGG (http://www.kegg.jp/). The top 30 genes enriched by a differently detected microbial community are presented in Figure 5 (*P* < 0.05). The function of a differently detected microbial community was close for PE and RE-PE, compared with RE-PU. In specific, RNA polymerase sigma, methyl-accepting chemotaxis protein, glutathione S-transferase, acetyl-CoA C-acetyltransferase, and acyl-CoA thioester hydrolase were rich in PE and RE-PE (Figure 5(a)). Gene families involved in fatty acid biosynthesis, glycolysis/gluconeogenesis, fatty acid degradation, glutathione metabolism, pyrimidine metabolism, fatty acid metabolism, iron complex outer-membrane receptor protein, RNA polymerase sigma-70 factor, ECF subfamily, and chromosome partitioning protein were significantly enriched in RE-PU and RE-PE, while those responsible for galactose metabolism, proteasome, starch, and sucrose metabolism were significantly associated with PE (Figure 5(b)).

## 4. Discussion

Combined periodontal-endodontic lesions, which can establish independently with or without communication between endodontic and periodontal components, are a kind of disease characterized by the interrelationship between the periodontal pocket and tooth pulp [22]. The prognosis of combined periodontal-endodontic lesions treated with traditional management is poor [23, 24]. Targeted therapy, aiming at the trigger event of combined periodontal-endodontic lesions, may have better performance and is eagerly needed. It is very important for clinicians to understand the pathogenesis process of combined periodontal-endodontic lesions.

The important role of microorganisms in oral diseases has been well recognized [20, 21, 25–28]. The specific mechanism of periodontal-endodontic lesions occurrence is still under debate; however, the important role of bacterial infection has been identified [6–8, 29, 30]. The transmission of microbial community and toxins between the periodontal pocket and the root canal was speculated to be the irritation [7]. Kipioti et al. found the similarity of flora between root canals and adjacent periodontal pockets of teeth with advanced periodontitis [6]. Kobayashi et al. also detected common microflora from root canals and periodontal pockets of caries-free teeth with advanced periodontitis [8]. Both of the studies above were conducted using the culture method only. Xia and Qi compared bacterial profiles using denaturing gradient gel electrophoresis (DGGE) between dental plaque and pulp of 13 teeth with combined periodontal-endodontic lesions [31]. However, the similarity of bacteria from dental plaque and pulp of the same tooth ranged from 13.1% to 62.5%. The conclusion might not be valid. Li et al. identified 43 genera/species from 20 patients' teeth with combined periodontal-endodontic lesions via DGGE [32]. The predominant genera were *Porphyromonas* sp. (13.9%), *Filifactor* sp. (12.5%), and *Parvimonas* sp. (11.1%). The most prevalent bacteria in the root canal and periodontal pocket were *Filifactor alocis*, *Parvimonas micra*, *Porphyromonas gingivalis*, and *Tannerella forsythia*. In general, previous studies revealed the possibility that the periodontal pocket was a source of root canal infection; however, due to the limited technique, the profile of differently distributed bacterial was not comprehensive. There was also a lack of follow-up research to elucidate the changing process after the root canal infection derived from the periodontal pocket.

It is interesting to note that not all teeth, which suffered from chronic periodontitis, will develop retrograde pulpitis and result in combined periodontal-endodontic lesions eventually. It is unclear which factor will decide the fate of the tooth. The present study showed the composition of the microbiota of RE-PE is differed from PE, indicating that different genera in periodontal pockets are the key to the occurrence of retrograde pulpitis. The fact that the microbiome of RE-PU was closer to RE-PE rather than PE further supported this viewpoint (Figure 1(c)). Highly rich genera in RE-PE may exert a pivotal role in the pulp invasion, leading to retrograde pulpitis. By LEfSe analysis, *Kingella* and *Barnesiella* are found to be highly rich in RE-PE than PE. *Kingella* is a genus of Gram-negative, aerobic, and facultatively anaerobic bacilli. *Kingella* is a normal flora in the oral cavity, being detected in both healthy and periodontitis subjects [33, 34]. To note, a study showed that *Kingella* was rich in biofilm collected from children with severe caries, indicating that *Kingella* might associate with invasion and erosion of enamel and dentin [35]. We speculate that predominate *Kingella* can degrade mineral substances and form the channel through which microbiota and secreted toxins can affect dental pulp. *Barnesiella* is reported to play an important role in several diseases as a major component of the gut microbiome [36–38]. A recent published study demonstrated that the abundance of *Barnesiella* would increase after *Porphyromonas gingivalis* infection, which was the etiological agent of periodontal disease [39]. It has been recognized that the gut microbiome is significantly associated with host immunity [40]. Thus, we speculated that *Barnesiella* may be able to affect the immune microenvironment of the periodontal pocket, damage the immune barrier, and accelerate the invasion of pathogenic microorganisms. The function of *Barnesiella* in periodontitis should be further investigated.

Previous studies showed that as the periodontal inflammation progresses, alteration of local flora might happen gradually [41–43]. To our knowledge, there is no study investigating the microbiota shift in retrograde pulpitis. The PCA analysis revealed that the composition of the microbiota of RE-PU is closer to PU rather than RE-PE. To note, all teeth diagnosed as combined periodontal-endodontic lesions are caries-free. Thus, the infection resource of retrograde pulpitis is only periodontal pockets. The significantly different microbiota composition between RE-PE and RE-PU indicates that the microenvironment has selected the microorganism by local pressures. The results of the alpha diversity analysis also support the assumption. The score of RE-PU is significantly lower than the PE and RE-PE groups, indicating that some predominant microorganisms are existing in the infected dental pulp.

The composition of microorganisms is significantly different among PU, RE-PE, and RE-PU. In detail, *Clostridiales Incertae Sedis XI*, *Parvimonas*, *Clostridium*, *Peptostreptococcaceae*, and *Filifactor* are more frequently detected in RE-PU, compared with PU and RE-PE. *Clostridiales Incertae Sedis XI* was created in 2001, and family members of *Clostridiales Incertae Sedis XI* were pathogen of septic arthritis, necrotizing pneumonia, chronic rhinosinusitis, and bacterial vaginosis [44–50]. This is the first time to illustrate the significant role of *Clostridiales Incertae Sedis XI* in oral disease. The function of *Clostridiales Incertae Sedis XI* is merit to be further explored. Delima et al. found that smoke cessation would lead to a decrease in the prevalence of *Parvimonas* [51]. The abundance of *Parvimonas* was proved to be rich in smokers with periodontitis than nonsmokers [52, 53]. *Clostridium* is a genus of gram-positive obligate anaerobes, including several significant human pathogens. Vigil et al. isolated *Clostridium* from 22 refractory periapical cases via growth culture [54]. Medina-Palacios et al. identified that *Clostridium* was one of the most common genera in refractory apical periodontitis [55]. One possible mechanism that *Clostridium* contributed to destruction is degrading Type IV collagen, which is the major component of basement membranes, via proteinases [56]. Several researchers have identified the abnormal accumulation of *Peptostreptococcaceae* in both chronic periodontitis and aggressive periodontitis [57–61]. In this study, we firstly reported *Peptostreptococcaceae* is rich in RE-PU. This adds a growing body of evidence that the periodontal pocket is the resource of infection for retrograde pulpitis. The role of *Peptostreptococcaceae* in pathogenesis of retrograde pulpitis needs to be further investigated. *Filifactor* is a genus of Gram-positive and anaerobic bacterium, containing *Filifactor alocis*, which is a diagnostic indicator of periodontal disease [62]. The most important characteristic of *Filifactor alocis* is the ability to survive in the oxidative stress-rich environment, especially periodontal pocket. Compared with *P. gingivalis*, *Filifactor alocis* is more resistant to H_2_O_2_-induced oxidative stress [63]. Moreover, it is interesting to note that the survival of *P. gingivalis* is significantly increased when cocultured with *Filifactor alocis*, indicating that *Filifactor alocis* may have the ability to detoxify the microenvironment and thereby improve the survival of pathogenic microorganism. Further study revealed the core role of “superoxide reductase” of *Filifactor alocis* in resistance to superoxide radicals [64]. Profound synergistic interactions among *Filifactor alocis* and oral bacteria have been confirmed by in vitro studies, suggesting the central role of *Filifactor alocis* in the microbial community of periodontal pocket [62, 63, 65, 66] and may majorly contribute retrograde pulpitis development.

Overall, this study is the largest cohort so far to investigate the microbiota for combined periodontal-endodontic lesions. The present study suggests that specific microbiota of periodontal pocket may be associated with the occurrence of retrograde pulpitis. The shift of microbial communities from periodontitis to retrograde pulpitis is investigated and revealed that the microenvironment of the root canal and pulp chamber can select predominate microflora. Due to the poor prognosis of retrograde pulpitis, more profound research and a better understanding of microorganisms might give us a hint on targeted medication to prevent the occurrence of retrograde pulpitis in the future.

## Figures and Tables

**Table 1:** The summary of grouping abbreviations.

Abbreviation	Grouping
PE	Chronic periodontics
PU	Ulcerative pulpitis
RE	Retrograde pulpitis
RE-PE	Periodontal pocket of retrograde pulpitis
RE-PU	Pulp of retrograde pulpitis

**Table 2:** Study population characteristics.

Characteristics	PU	PE	RE
Gender			
Male	8	9	6
Female	15	5	5
Age (mean, range)	36.7 (12-66)	52.2 (38-76)	53.9 (45-62)
Location			
Anterior tooth	4	2	—
Premolar	5	4	—
Molar	15	12	11
Systematic conditions			
Hashimoto's disease	1	—	—
Hypertension	1	3	2
Diabetes	—	3	2

## Data Availability

The data that support the findings of this study are available from the corresponding authors upon request.

## Supplementary Materials

Supplementary Materials Figure S1: the Venn diagram contouring the distribution of operational taxonomic units (OTUs) for (a) PE, RE-PE, and RE-PU and (b) PU, RE-PE, and RE-PU.
