# Supplementary figures and images for: The Microbiota Profile Analysis of Combined Periodontal-Endodontic Lesions Using 16S rRNA Next-Generation Sequencing
Source: J Immunol Res. 2021 Nov 16;2021:2490064. doi: 10.1155/2021/2490064 (PMC8610669; doi:10.1155/2021/2490064)

## Slide 1
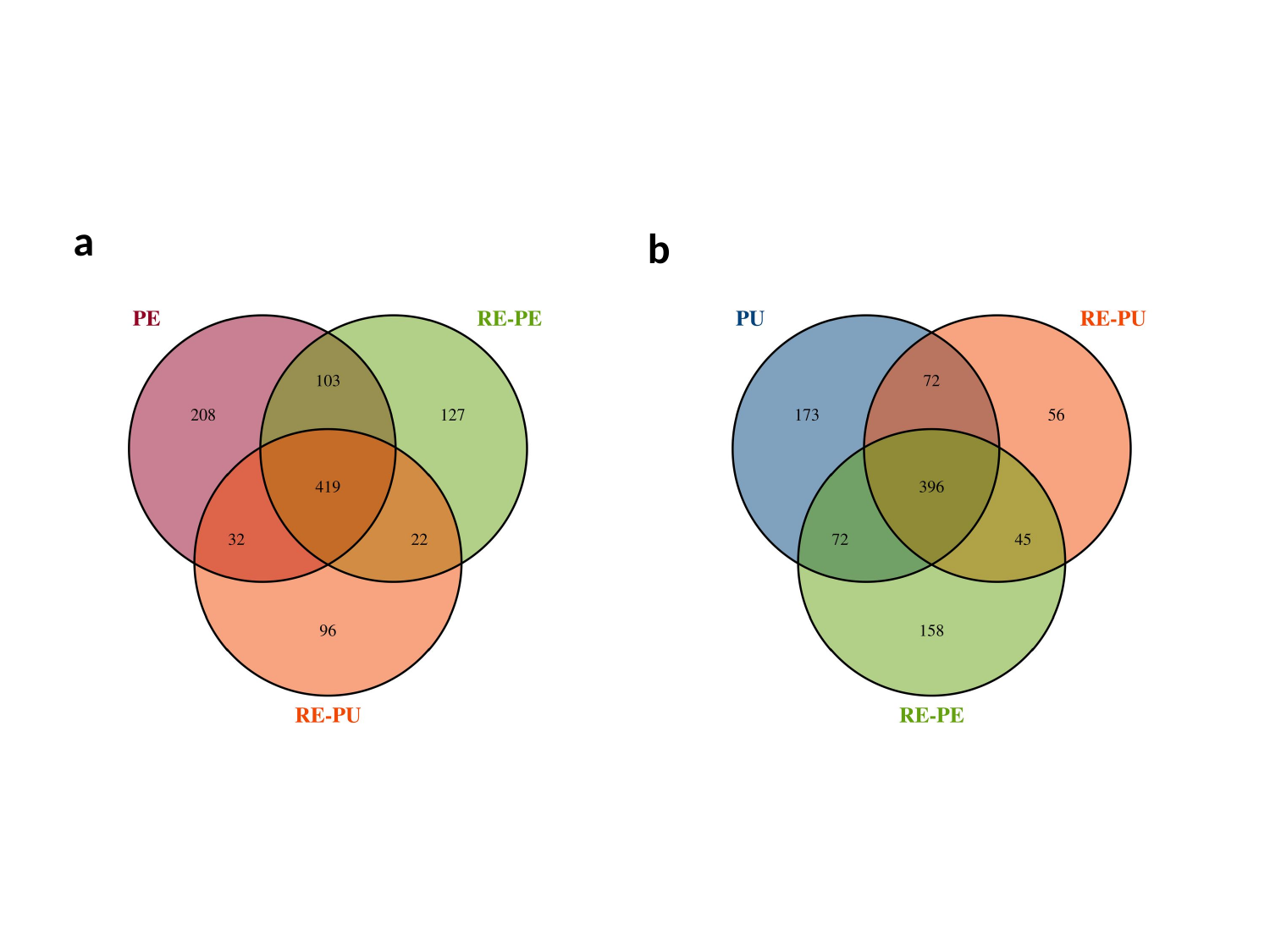

a
b

Supplement: Supplementary Materials — Figure S1: the Venn diagram contouring the distribution of operational taxonomic units (OTUs) for (a) PE, RE-PE, and RE-PU and (b) PU, RE-PE, and RE-PU. [file 2490064.f1.pptx]
